# Identification of Novel *IL-10RA* Variant in Infantile‐Onset Inflammatory Bowel Disease: A Case Series With Preliminary Genotype–Phenotype Correlation From Two Chinese Families

**DOI:** 10.1155/carm/6654628

**Published:** 2025-12-15

**Authors:** Chengcheng Guan, Yuanxuan Ma, Xiao Zhang, Ru Zhang, Yingjun Jiang, Shiguo Liu

**Affiliations:** ^1^ Medical Genetic Department, The Affiliated Hospital of Qingdao University, Qingdao, Shandong, China, qdu.edu.cn; ^2^ The Prenatal Diagnosis Center, The Affiliated Hospital of Qingdao University, Qingdao, Shandong, China, qdu.edu.cn; ^3^ Gastrointestinal Surgery, The Affiliated Hospital of Qingdao University, Qingdao, Shandong, China, qdu.edu.cn

**Keywords:** genotype and phenotype relationships, infantile-onset inflammatory bowel disease, interleukin-10, IL-10RA mutations, whole-exome sequencing

## Abstract

Interleukin‐10 (IL‐10) signaling is critical for intestinal immune homeostasis, and defects in this pathway underlie infantile‐onset inflammatory bowel disease (IOIBD). While numerous IL‐10‐related mutations have been reported, their pathogenic mechanisms and genotype–phenotype correlations remain incompletely understood. Here, we describe four patients from two Chinese families with IOIBD harboring novel IL‐10RA mutations. We detailed the clinical presentations and performed bioinformatics analyses to assess mutation conservation, pathogenicity, and structural impact via 3D modeling. Our findings broaden the IL‐10RA mutation spectrum and provide preliminary genetic insights that may support clinical diagnosis and prenatal screening for IOIBD in Chinese populations, while also highlighting the need for further functional validation.

## 1. Introduction

Inflammatory bowel disease (IBD) is a chronic gastrointestinal disease induced by multiple factors, such as environment, inflammation, intestinal flora, genetic background, and so on, and can be divided into different subtypes including Crohn’s disease (CD), ulcerative colitis (UC), and indeterminate colitis (IC) [1]. Different from adult‐onset IBD in disease phenotype, the pediatric IBD (PIDB) may present the above three disease’s features and further be divided into several different subgroups based on the age of onset and clinical features, among which very early‐onset IBD (VEOIBD) refers to the onset before 6 years, accounting for about 6%–15% [2, 3]. In addition, patients with onset earlier than 2 years of age belong to the infantile form of IBD (IOIBD), which accounts for approximately 1% of patients with PIDB [4].

The typical manifestations of VEOIBD present repeated fever, ineffective for immunosuppressive therapy, and severe gastrointestinal symptoms including colitis, perianal abscess, and fistulae [5–7]. Compared with adult‐onset IBD, the differences in VEOIBD are mainly as follows: (1) the lesion limited to the colon, (2) resistant to standard treatments for IBD, and (3) high association with genetic susceptibility [8]. Besides, VEOIBD patients may show a higher likelihood of rectal bleeding [6]. As an increasing number of refractory and severe VEOIBD cases were considered to be Mendelian disease, more than 50 candidate genes such as interleukin‐10 (*IL-10*)‐related genes, *XIAP*, and *FOXP3* were associated with the pathogenesis of VEOIBD [2, 9, 10]. Particularly, the role of *IL-10*‐related genes has attracted more and more attention due to early disease onset, poor treatment effect, and early surgical treatment for infant VEOIBD.

As an anti‐inflammatory cytokine, IL‐10 plays an essential role in mucosal immunity through binding to IL‐10 receptor (IL‐10R) composed of two IL‐10 receptor *α* (IL‐10RA) subunits and two IL‐10RB subunits. Mutations in *IL-10* and *IL-10RA/B* have been reckoned as the primary cause of IOIBD [2, 11, 12], characterized by severe perianal infection and folliculitis, resistant to conventional treatments, and effective in hematopoietic stem cell transplantation (HSCT) [3, 13, 14]. *IL-10RA* (OMIM: 146933), located on the position of chromosome 11q23, consisted of 8 exons and encoded a 578‐amino‐acid protein. As the IL‐10RA protein can bind to IL‐10 to promote the interaction between IL‐10 and IL‐10B through the conformational change of IL‐10RB [13], the heterotetramer composed of IL‐10RA and IL‐10RB could further activate downstream signaling pathways and mediate the anti‐inflammatory role [15]. In the intestinal environment, hundreds of millions of intestinal flora constantly stimulate the immune system, where IL‐10RA plays an anti‐inflammatory and immunosuppressive role to maintain the balance of intestinal immunity. An animal experiment also found that the mice with knocked‐out *IL-10/IL-10R* exhibited a series of chronic enteritis–like symptoms: recurrent diarrhea, growth retardation, intestinal mucosal hyperplasia, and inflammatory response, suggesting *IL-10/IL-10R* may be involved in the pathogenesis of IBD [16]. *IL-10RA* mutations in IOIBD were more common in Asia compared with America and Europe, and there was a cohort study on the relationship between genotype and phenotype in China; however, the relationship between genotype and clinical phenotype remains unclear [17]. Here, we performed a genetic study in two unrelated IOIBD families, further predicted the pathogenicity of four gene mutations identified in these two families, and analyzed the association between genotype and phenotype. Our study enriched the genetic mutation and clinical phenotype profile of the VEOIBD, laying the foundation for more accurate diagnosis and individualized treatment.

## 2. Materials and Methods

### 2.1. WES

Genomic DNA was extracted from the peripheral blood using the Qiagen DNA Extraction Kit (QIAGEN). DNA concentration was quantified using a spectrophotometer (Thermo Fisher Scientific Oy Retaste 2, FI‐01620 Vanta). For WES library preparation, qualified DNA was fragmented into sizes ranging from 100 to 700 bp using sonication (Covaris S2, Covaris). The libraries were then verified using NanoDrop 2000 and agarose gel electrophoresis (TSINGKE). Target genes were captured using biotin‐labeled probes and specific magnetic beads. The captured target genes were subsequently absorbed by a magnetic frame, washed, and purified to enrich the target sequences. The qualified enriched libraries were sequenced on an Illumina NextSeq 500 sequencer (Illumina) for paired‐end 150‐bp reads. After sequencing, low‐quality variants were filtered using a quality score threshold of ≥ 20. The filtered reads were aligned to the reference human genome (hg19) using BWA. False‐positive single‐nucleotide polymorphisms (SNPs) around insertions or deletions (InDels) were eliminated. The pathogenicity of the identified variants was predicted using several bioinformatics software tools.

### 2.2. Sanger Sequencing Verification

Following the identification of highly suspected pathogenic gene mutations through WES, the upstream and downstream sequences of the mutation sites were amplified by PCR and subjected to Sanger sequencing. Primers were designed using Primer Premier 5 software (forward: GGC​GAG​CTC​GTG​AGA​TAC​CT; reverse: ACC​CCC​AAC​TTT​TGC​TTG​GT for ADAR1 c.2668G > C). The PCR‐amplified products were analyzed using 1% agarose gel electrophoresis (TSINGKE), and the purified products were sequenced on an ABI 3730 analyzer (Applied Biosystems). A comparison with the National Center for Biotechnology Information (NCBI) database (https://www.ncbi.nlm.nih.gov/) revealed a novel mutational site in the proband.

### 2.3. Genetic and Bioinformatics Analysis

DNA sequences were obtained from EditSeq, and Chromas was used to interpret gene sequencing profiles. Gene sequence alignment and mutation site localization were performed using SnapGene and the BLAST function of the NCBI website. The pathogenicity of missense mutations was analyzed using PolyPhen‐2, PROVEAN, and FruitFlySplice predictor. DNAMAN software was used to compare and analyze the conservation of the *IL-10RA* sequence, with SWISS‐MODEL used to construct a 3D structural model of the IL‐10RA protein.

## 3. Case Presentation

### 3.1. Clinical Manifestations

Patient in family A (AII2), a six‐month‐old baby girl, has recurring diarrhea and fever with no obvious cause since birth, and stool was discharged from the perineum for 4 months. This child was delivered smoothly without a history of birth injury or asphyxia. Since the onset of the disease, the patient has a good state of mind, good diet, and sleep but poor weight gain, yellow‐green watery stools, and normal urination. She had an older brother diagnosed with chronic diarrheal disease, cytomegalovirus infection, and septicopyemia and died at 6 months old. Physical examination showed an increased pulse of 100 beats/min and a weight of 5 kg. The perianal and perineal areas were obviously red and swollen with ulcerated surface skin, and multiple fistulas can be seen. There was a polyp with a diameter of 0.5 × 0.5 cm in the perianal area. Besides, limb muscle strength can be discoordinated, muscle tension was low, and both lower limbs were slightly swollen. Abdominal ultrasound showed inflammatory changes in the colon, multiple small ulcers, no signs of intussusception, intestinal obstruction, and perforation. Anal ultrasound indicated that a fistula was formed in the soft tissue of the perineum, connecting the anal canal, and there was gas echo in the soft tissue between the rectum and vagina, suggesting rectal–vaginal connection. Bone marrow puncture showed that erythroid hyperplasia is stagnant, and the granulocyte/erythrocyte ratio was increased. Meropenem 0.1 g per 8 h was injected for anti‐infection treatment, and Smecta and *Lactobacillus acidophilus* were used for protecting the gastrointestinal mucosa and regulating intestinal flora. Considering the patient’s decreased albumin, albumin was infused to provide nutritional support. However, the child’s family finally gave up treatment.

Patient BII2 in family B was 8 months old and had recurrent chronic diarrhea with repeated fever since 1 month of birth. Besides, he has developed anal mass hyperplasia with pus outflow for 4 months. During breastfeeding, the infant was in a suboptimal mental state but demonstrated no signs of resistance. Stool traits were yellow and watery. This child has suffered from eczema since birth and was born at full term with no history of asphyxia or birth injury. He also had an elder brother who was diagnosed with chronic diarrhea and severe malnutrition with weight loss and died 3 years ago at the age of 7 months. The patient presented with a malnourished appearance and a moderate physical development. The vital signs including temperature, pulse, blood pressure, respiratory rate, and weight were, respectively, 38.3°C, 144 beats/min, 100/60 mmHg, 42/min, and 6 kg. He had thin subcutaneous fat, poor skin elasticity, and faded brownish rashes on his face and front chest. A physical examination of the head, neck, and face showed no abnormalities. The thorax was symmetrical without deformity. Breathing sounds in both the lungs were clear and no murmur, but the respiratory rate increased to 44/min. The heart rate was up to 144 beats/min, and the heart rhythm was regular without a heart valve murmur. A hyperplasia of mass with pus flowing out of the ulcer was found at the back left of the anus. Considering that the baby was too small, the parents refused to do a colonoscopy. Adenosine monophosphate and cefuroxime sodium were used for anti‐infection treatment. Rehydration was performed to provide nutritional support to the patient. Besides, we used montmorillonite powder for diarrhea. The patient’s symptoms recurred with supportive treatment and eventually died after 1 month.

**Figure 1:**
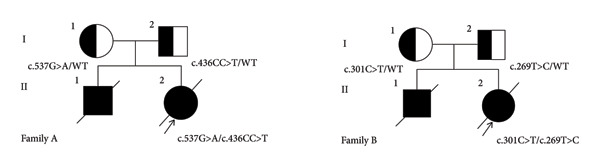
Family diagram of families A and B.

We performed laboratory examinations on two patients, and the consistent abnormal results are listed in Table 1. There was no abnormality in fecal tests of the flora analysis, bacterial culture, and rotavirus and adenovirus antigen detection in both the patients. However, unlike patient BII2’s normal stool routine results, patient A’s stool routine showed excessive white blood cells. Inconsistency also reflected in the results of regulatory lymphocyte subsets, with a decrease in total T cells, suppressor T cells, and resting T cells in patient AII2 and an increase in patient BII2. In addition to these test items, the two patients have specific abnormal indicators, respectively. In patient AII2, the results of the coagulation routine revealed an increase in APTT and D‐dimer and a decrease in antithrombin III. Besides, IBD‐related autoantibodies were negative. In patient BII2, the indicators of creatine kinase‐MB, lactate dehydrogenase, and high‐sensitivity troponin were elevated (Figure 1).

**Table 1:** Laboratory tests with abnormal results in both patients.

	Patient AII2	Patient BII2	Reference interval
WBC	↑	↑	5.0–12.0 (n/L)
NEUT	↑	↑	25.0–55.0 (%)
MONO	↑	↑	40.0–70.0 (%)
RBC	↓	↓	3.7–5.5 (n/L)
Hb	↓	↓	110.0–145.0 (g/L)
HCT	↓	↓	40.0–50.0 (%)
CRP	↑	↑	0.0–5.0 (mg/L)
PLT	↑	↑	100.0–300.0 (n/L)
TP	↓	↓	65.0–85.0 (g/L)
Alb	↓	↓	35.0–55.0 (g/L)
PA	↓	↓	200.0–400.0 (mg/L)
HDL	↓	↓	0.8–1.8 (mmol/L)
LDL	↓	↓	1.9–3.1 (mmol/L)
BUN	↓	↓	3.1–8.0 (mmol/L)
GA	↓	↓	10.4–15.7 (%)
SA	↑	↑	35–55 (g/L)
TBA	↑	↑	0.0–10.0 (μmol/L)
*α*1GLB	↑	↑	1.5–3.5 (%)
*α*2GLB	↑	↑	6.0–10 (%)
ALT	↑	↑	7.0–40.0
AST	↑	↑	13.0–35.0
IgE	↑	↑	0–15 (IU/mL)
CD4/CD8	↑	↑	0.9–1.9
Activated B + NK cells	↑	↑	3.0–11.7 (%)

### 3.2. Genetic Results

**Figure 2:**
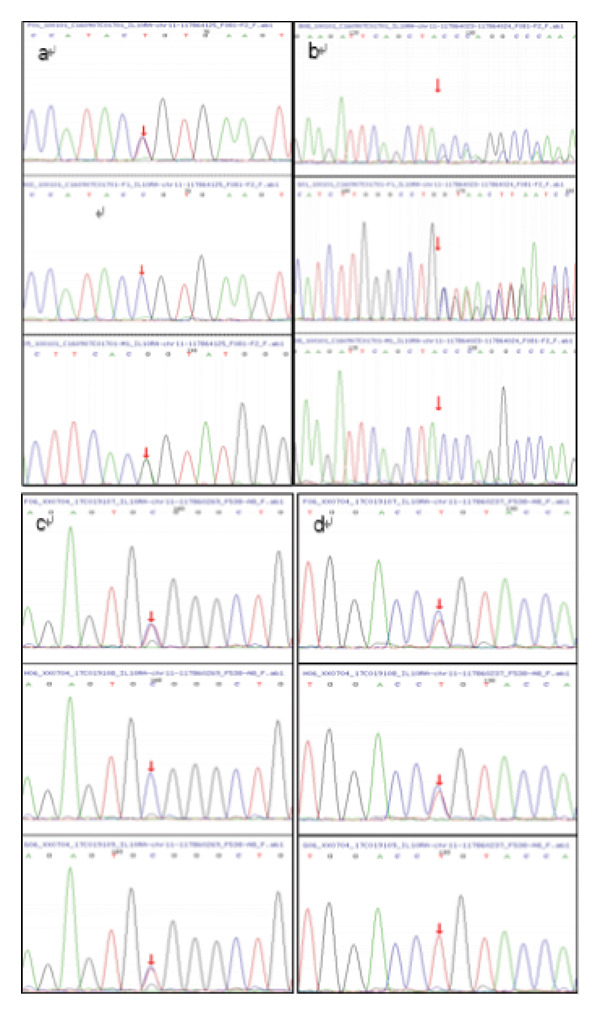
Sequencing chromatograms of the *IL-10RA* variants. (a) The sequencing results of the c.537G > A mutation of the patient and her father and mother in family A; (b) the sequencing results of the c.436CC > G mutation of the patient and her father and mother in family A; (c) the sequencing results of the c.269T > C mutation of the patient and his mother and father in family B; (d) the sequencing results of the c.301C > T mutation of the patient and his mother and father in family B.

We, respectively, found compound heterozygous mutations of IL‐10RA in two patients from unrelated families through WES (Figure 1). The mutations were c.537G > A and c.436CC > G in family A and c.269T > C and c.301C > T in family B, among which c.436CC > G was a novel mutation, neither found in ExAC nor 1000G (Table 2). After verification by Sanger sequencing, we identified that the mutation c.537G > A is inherited from the patient’s mother and another mutation c.436CC > G is carried by her father in family A (Figures 2(a) and 2(b)). In family B, mutations c.269T > C and c.301C > T were, respectively, inherited from the patient’s father and mother (Figures 2(c) and 2(d)). Furthermore, the deceased brothers of the two probands both exhibited similar clinical symptoms. This familial history provides compelling clinical support for the pathogenicity of the variant under investigation. Although direct genetic confirmation in the deceased individuals was not feasible, this observation strongly suggests an association between the variant and the familial clustering of the disease. For mutation c.537G > A, the substitution from G to A is located on exon 4, causing a synonymous mutation (p.T179 = ). Another mutation c.436CC > G is also located on exon 4, belonging to a frameshift mutation (p. 146Pfs∗40), caused by change in the amino acid from Pro to Ala, encoding termination at position 40 after the mutation site. The mutations c.301C > T and c.269T > C located on exon 3, respectively, occurred due to the change in the amino acid from Arg to Trp (p.R101W) and from Leu to Pro (p.L90P), both belonging to a missense mutation. The positions in IL‐10RA of four mutations are shown in Figure 3.

**Table 2:** Comprehensive variant annotation details.

	c.537G > A	c.43CC > G	c.301C > T	c.269T > C
HGVS	NM_001558.4:c.537G > A; NP_001549.2:p.Thr179 =	NM_001558.4:c.43CC > G; NP_001549.2:p.146Pfs∗40	NM_001558.4:c.301C > T; NP_001549.2:p.Arg101Trp	NM_001558.4:c.269T > C; NP_001549.2:p.Leu90Pro
ClinVar ID	830051	_	39432	_
dbSNP ID	rs1419560997	_	rs368287711	_
OMIM ID	_	_	146933.0005	_
ACMG	Pathogenic	Pathogenic	Likely pathogenic	Uncertain
ExAC (gnomAD)	0.00001	_	0.000007525	_
1000G	_	_	0.00020	_

*Note:* Four variants were annotated from public databases (dbSNP, ClinVar, OMIM) following HGVS nomenclature. ACMG is used for predicting the pathogenicity of variants. Population allele frequencies are presented as percentages from the ExAC(gnomAD) and 1000 Genomes Projects. “_”, no described.

Figure 3Multilinear homology comparison between different species. (a) The conservative L90, R101 in six mammalian species and (b) the conservative P146 in six mammalian species.

**Figure:**
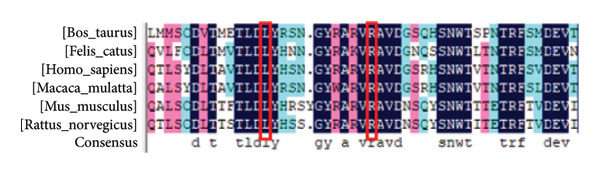
(a)

**Figure:**
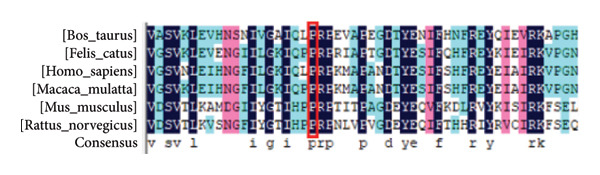
(b)

### 3.3. Bioinformatics Results

**Figure 4:**
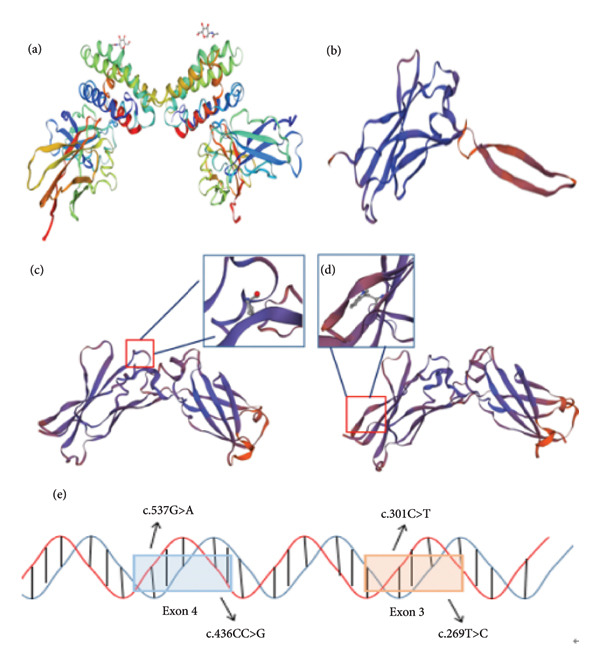
Impact of the variants on the structure of the MAN2B1 protein. (a) The IL‐10RA protein structure; (b) the protein structure of IL‐10RA with mutation c.436CC > G; (c) the protein structure of IL‐10RA with mutation c.261T > C; (d) the protein structure of IL‐10RA with mutation c.301C > T; and (e) the locations of the four mutations in the *IL-10RA*.

To evaluate the conservative mutation sites, we used DNAMAN software to compare the amino acid sequence of nonsynonymous mutations in different mammalian species (Figure 3). As shown in Figure 3, c.269T > C (p.L90P), c.301C > T (p.R101W), and c.436CC > T (p.146Pfs∗40) were highly conserved. The frameshift mutation of c.436CC > T in family A was predicted by MutationTaster to be disease‐causing, leading to premature termination of amino acid coding, which may induce the nonsense‐mediated mRNA decay. Another mutation c.537 G> A is located at the last base position on exon 4, so we inferred that this mutation may cause a change in the splicing site. FruitFlySplice Predictor predicted this site as the donor of the splicing site, with a score of 0.92 (cutoff 0.40). Besides, the score in Alternative Splice Site Predictor was 8.047, higher than the cutoff of 4.5, so the c.537 G> A mutation was presumed to be the donor site. For family B, PROVEAN and PolyPhen‐2 were used to predict the pathogenicity of missense mutations found in the patient, and the risk values were considered to be lower than −2.5 and close to 1, respectively. The mutation c.269T > C was reckoned as a pathogenic mutation, with a score of 0.977 for PolyPhen‐2 and −3.036 for PROVEAN. The score of c.301C > T was predicted as 1 by PolyPhen‐2, indicating a highly pathogenic correlation. Besides, the score of PROVEAN was −6.472. Then, we constructed the protein 3D model of three nonsynonymous mutations, in which mutation c.436CC > G clearly led to a truncated protein (Figure 4).

## 4. Discussion

With the wide application of sequencing technology, more and more VEOIBD were considered to be monogenic disorders due to the detection of gene mutations. The single genetic mutation is more likely to involve the pathogenesis of VEOIBD in infancy than in older children [3, 9]. It has been proved that monogenic defects can cause intestinal homeostasis imbalance through multiple mechanisms, such as epithelial barrier impairment, immune cell defects, and immunomodulatory disturbance, and the mutations in IL‐10 signal pathway lead to a decreased immunosuppressive function, further mediating the excessive activation of the immune response [2]. Because signal transducer and activator of transcription 3 (STAT3) is an important transcription factor of the IL‐10 signal pathway, the degree of phosphorylated STAT3 can reflect the IL‐10 signal pathway. A deficiency of phosphorylated STAT3 was observed in peripheral blood mononuclear cells (PBMCs) cultured with *IL-10* with heterozygous mutations, indicating that the defects in *IL-10*‐related genes may affect the function of IL‐10 and silence its signaling pathway [18]. Besides, a recent study indicated that IOIBD patients with *IL-10*‐related gene mutations showed a higher clonality of the B‐cell receptor and T‐cell receptor repertoire in peripheral blood lymphocytes, and this unique increase may be implicated in the tissue damage [19].

Glocker EO et al. first described in 2009 that mutations in *IL-10R* were related to the onset of IOIBD and subsequently detected additional *IL-10*‐related gene mutations in patients with VEOIBD [20]. Here, we identified four mutations of *IL-10RA* (c.269T > C, c.301C > T c.436CC > T, and c.537G > A) in two IOIBD patients from unrelated families, of which c. 301C > T and c.537G > A were hotspot mutations in Asian population [4, 21], but c.436CC > G is a previously unreported mutation. The three known mutations have been described by previous studies. Under the stimulation of lipopolysaccharide, the PBMC secreted higher levels of cytokines in an IOIBD patient carrying with IL‐10RA compound heterozygous mutations (c.251C > T/c.301C > T) than in healthy individuals, and this increase was not inhibited by IL‐10 [22]. The mutation *IL-10RA* (c.537G > A), a mutation that is a common cause of the disease, was first identified in a Japanese infant by Tadahiro Yanagi who suggested that this mutation leads to the production of at least two abnormal transcripts and further loss of IL‐10R function [23]. For mutation *IL-10RA* (c.269T > C), the first description was shown in a 7‐month‐old baby girl with the manifestation of rectovaginal fistula [24]. The novel mutation *IL-10RA* (c.436CC > T) found in this study was initially confirmed as a pathogenic mutation, and whether it affects the expression and function of IL‐10 remains to be further studied. In addition to these discovered point mutations, the deletion of gene fragments in exon 1 of *IL-10RA* has also been proved to be related to the occurrence of IOIBD [24].

IOIBD mediated by *IL-10/IL-10R* mutations usually presented as a more malignant disease than VEOIBD, mainly due to an early age of onset, inadequate treatment response, and poor prognosis. Through a review and study of multiple literatures [11, 17, 24, 25], we have summarized several of the most common clinical symptoms of this disease, including diarrhea, perianal lesions, bloody stool, growth retardation, oral ulcers, skin rashes, and folliculitis. In our study, both patients developed diarrhea, perianal disease, and growth retardation, while patient A also suffered from a rash (Table 3). Hearing impairment exhibited by patient AII is a rare symptom of the disease that only three patients were with this symptom in a retrospective study including 139 IOIBD patients [17]. Both patients had elevated platelet counts, but a Japanese IOIBD patient with c.537G > A homozygous mutation had thrombocytopenic purpura [23], reminding us that there is a difference effect to phenotype between homozygous and heterozygous. Besides, comparing clinical data from VEOIBD patients with *IL-10/IL-10R* mutation and those without the gene mutation revealed that the former had significantly slower weight gain and affected growth and development in children [25]. Consistent with these results, the two patients in our study showed delayed growth that the body weight and serum albumin were both lower than the lower limit of the reference value. Apart from the above symptoms, B‐cell lymphoma has been reported in patients with IL‐10 signaling deficits, and interestingly, B‐cell lymphoma had a higher incidence in *IL-10RB* mutation patients than in *IL-10RA* mutation patients [17].

**Table 3:** Comparison of clinical characteristics of two patients.

	Patient A	Patient B
Gender	F	M
Age of onset (months)	1	1
Family history	+	+
Diarrhea	+	+
Repeated fever	+	+
Perianal lesions	+	+
Bloody stool	−	−
Growth retardation	+	+
Oral ulcers	−	−
Skin rashes	−	+
Myocardial damage	−	+
Liver damage	+	+
Hearing impaired	+	−

At present, HSCT is considered the only effective treatment for IOIBD attributed to *IL-10/IL-10R* mutations. Daniel et al. [26] demonstrated that the *IL-10* gene signaling pathway was reconstituted in patients after receiving HSCT. A retrospective study indicated that there were five patients who achieved clinical remission, accounting for 71.4% of the total patients receiving cord blood stem cell transplantation [24]. After umbilical cord blood transplantation was performed in nine patients, six patients were completely relieved and the symptoms of malnutrition and growth retardation improved [27]. However, Cuifang et al. [17] raised their concerns that although this treatment was indeed effective, the death caused by its complications should not be underestimated. For the diagnosis of IOIBD, it is essential to perform genetic testing to determine the cause. Additionally, given the similarity in intestinal disease phenotypes, the differentiation between IOIBD and food protein‐induced allergic proctitis should be carefully considered. Recently, serum ferritin was identified as a valuable indicator of association with Mendelian IOIBD, and IL‐10 levels and age of onset were positively correlated with mutation‐induced IOIBD in *IL-10*‐related genes [4].

## 5. Conclusion

Here, we identified four *IL-10RA* mutations leading to IOIBD, of which c. 436CC > G was a novel mutation, and analyzed their pathogenicity and impact on the protein structure. Subsequently, we may further explore the effects of novel mutation c.436CC > G on IL‐10 expression and signaling pathways through in vitro experiments. Besides, our study reviewed and analyzed the clinical features of two patients and reported cases, comparing the consistency and differences. In summary, we enriched the mutation and phenotype spectrum of IOIBD caused by *IL-10/IL-10R* mutations, contributing to the etiology and treatment of the disease.

## Ethics Statement

The study was approved by the Ethics Committee of the Affiliated Hospital of Qingdao University, Ethics Approval No. QYFY WZLL 27300.

## Disclosure

All authors read and approved the final manuscript to be published and agreed to be responsible for the accuracy of the data and details.

## Conflicts of Interest

The authors declare no conflicts of interest.

## Author Contributions

Liu proposed the idea and concept of the study and designed the plan for the case. Guan and Ma performed data collection and analysis. Xiao Zhang and Ru Zhang drafted and revised the manuscript.

## Funding

This work was supported by National Key Research and Development Program (grant numbers 2016YFC1000306 the National Natural Science Foundation of China (NSFC) (81371499), and Qingdao Scientific Technology Demonstration Project of Benefiting the People (24‐1‐8‐smik‐5‐nsh).

## Data Availability

The data that support the findings of this study are available on request from the corresponding author. The data are not publicly available due to privacy or ethical restrictions.
